# A light-driven device for neuromorphic computing

**DOI:** 10.1038/s41377-024-01722-9

**Published:** 2025-01-08

**Authors:** Shimul Kanti Nath

**Affiliations:** School of Photovoltaic and Renewable Energy Engineering, University of New South Wales (UNSW Sydney), Kensington, NSW Australia

**Keywords:** Optical data storage, Optoelectronic devices and components

## Abstract

A unique optoelectronic synaptic device has been developed, leveraging the negative photoconductance property of a single-crystal material system called Cs_2_CoCl_4_. This device exhibits a simultaneous volatile resistive switching response and sensitivity to optical stimuli, positioning Cs_2_CoCl_4_ as a promising candidate for optically enhanced neuromorphic applications.

The human brain is an extraordinary computer capable of processing fault-tolerant information and making decisions based on dynamic and complex situations. Unlike conventional computers, which struggle with processing probabilistic, noisy, and inconsistent data, the brain performs these tasks with remarkable accuracy and power efficiency. Central to its superior computing abilities are neurons and synapses (Fig. 1a), the basic units responsible for transmitting and processing data and information. Each neuron interacts with thousands of others, creating a massively parallel and complex network, while synapses adjust the strength of signals traveling between neurons, combinedly facilitating critical processes like learning, memory, recognition, and decision-making.

**Fig. 1:**
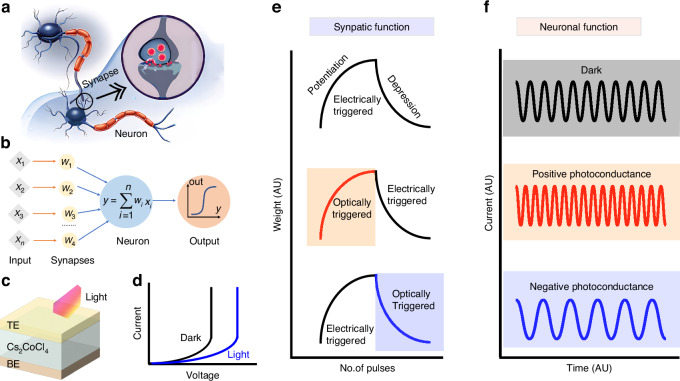
Schematics of (**a**) biological neurons and synapse, (**b**) artificial neural network, (**c**) device structure (TE and BE represent the top electrode and bottom electrode, respectively), (**d**) volatile threshold switching, (**e**) synaptic functionality showing electrically and optically triggered potentiation and depression with different scenarios, and (**f**) neuronal functionality (spiking dynamics) controlled by positive and negative photoconductance effects

Inspired by the cognitive prowess of the brain, a new generation of technology, namely artificial synapses and neurons, has emerged in recent years^1,2^. These are used to construct artificial neural networks, as illustrated in Fig. 1b, where they replicate the behavior of biological counterparts. To create artificial synapses and neurons, an insulating or semiconducting layer is often placed between two metal electrodes, making a resistive switching device, now frequently called a memristor, as shown in Fig. 1c. Their core functionality lies in altering electrical resistances, which can be permanent or transient depending on the choice of materials, device geometry, and operating conditions.

Many artificial synaptic and neuronal devices are based on oxides, chalcogenides, phase-change materials, and perovskites^3–5^. They primarily rely on electrical stress to process, store, and erase information. Specifically, synaptic functions such as adjusting synaptic weights (termed as ‘potentiation’ and “depression”), as well as mimicking the spiking behavior of neurons (see Fig. 1e, f, respectively), are often achieved by applying voltage or current pulses. With the emergence of artificial intelligence and neurorobotics, a great interest has grown in developing devices that can also respond to optical stimuli. This dual responsivity is highly advantageous for image and pattern recognition, as well as in-sensor and near-sensor computing. It offers cost-effectiveness and energy efficiency by eliminating the need for external photo (light) sensors to capture visual (or optical) information, thus simplifying device architecture and reducing latency, material consumption, and processing steps. Despite these promising prospects, only a few material systems have demonstrated efficient optical response while maintaining reliable resistive switching properties, and many of them are only sensitive to a narrow band of electromagnetic spectrum such as ultraviolet illumination. This limitation greatly hinders the development of optoelectronic neuromorphic technology.

In a conventional optoelectronic synaptic device, optical stimulation is used to trigger potentiation (a gradual increase in device conductance), and electrical stimulation is applied to induce synaptic depression (gradually restoring the device to its previous conductance or resistance state), as depicted in the middle panel of Fig. 1e. This is possible due to the positive-photoconductance property of the photoactive material, where the device conductance increases with optical illumination. Conversely, achieving optically triggered synaptic depression (bottom panel of Fig. 1e) is more challenging, often requiring materials with negative-photoconductance characteristics. Unfortunately, memristor devices offering reconfigurable resistance states and negative photoconductivity are relatively scarce, making such devices a hurdle in the research field.

Addressing this scarcity, Huifang Jiang and colleagues from Zhengzhou University and Beijing Institute of Technology have introduced an exciting material system: cesium cobalt chlorine (Cs_2_CoCl_4_) in the single-crystal form that can be used as an optoelectronic synaptic device^6^. Their findings, published in Light: Science & Applications, show simultaneous volatile resistive switching and negative photoconductance, making it a unique material system to exhibit such a response. The authors demonstrated a Cu/Cs_2_CoCl_4_/ITO device that shows synaptic functions mediated by electrically triggered Cu migration (potentiation) and negative photoconductance of Cs_2_CoCl_4_ (depression). Notably, the device is sensitive to broad optical stimuli, ranging from 265 to 780 nm (ultraviolet to visible light), and demonstrates a highly efficient specific detectivity on the order of 10¹² Jones, which makes it a promising candidate for processing a broad range of optical information. Through extensive experimental and theoretical analyses, the authors identified that the negative photoconductance response arises from the trapping of photo-excited electrons in Cs_2_CoCl_4_. The authors further demonstrated the practical application of their device by constructing a simulated artificial neural network, which successfully recognized handwritten digital images with high accuracy.

A notable feature of the reported device is its volatile threshold switching response, where the threshold voltage can be engineered using optical illumination, as illustrated in Fig. 1d. Remarkably, this characteristic persists even when the Cu electrode is replaced with an inert Au electrode, indicating that the volatile switching mechanism is primarily governed by the conduction in Cs_2_CoCl_4_. Note that the resistive switching in devices with Cu or Ag electrodes is mainly governed by the electric field-induced migration of metal ions, and the dielectric material plays a passive role in the conduction process. The authors have highlighted that the negative photoconductance of Cs_2_CoCl_4_ increases the threshold voltage of the device due to the increase of overall device resistance, in contrast to the behavior of positive photoconductance-based volatile switching devices, where the threshold voltage typically decreases^7^. These unique properties open new pathways for developing advanced neuromorphic devices that integrate electrical and optical functionalities, promising more versatile, cost- and energy-efficient systems for future applications such as artificial intelligence, machine vision, and neuro-robotics^8^. This device can also be used beyond neuromorphic computing, including as an access device (selector) for ReRAM-based digital memories, true random number generator, creating physical unclonable functions for application in information security, etc.^9^.

One foreseeable application of this device is the implementation of a light-tuneable artificial neuron that operates via frequency modulation, as schematically shown in Fig. 1f. This neuron could be utilized for in-sensor spike encoding, which is helpful for image segmentation, pattern recognition, and motion detection^7,10–12^. Similarly, it can also be used as a thermoreceptor by harnessing temperature-dependent threshold switching characteristics of the device^13^, which might be useful for applications such as robotic touch and human-machine interaction. As this study advances the growing field of photo-tunable volatile memristive switching devices, it opens up several future research opportunities. These include exploring the reliability and reproducibility of such characteristics at the nanoscale, expanding sensitivity to the near-infrared and short-wave infrared spectrum through bandgap or defect engineering, improving the linearity of synaptic potentiation and depression through materials and interface engineering, and studying frequency tunability in artificial neurons under both electrical and optical stimuli, to name a few.

Overall, this work offers a fresh perspective on exploring new materials and strategies for developing optoelectronic devices with cognitive abilities. By adopting this material, we are moving closer to mimicking biological neurons and synapses using a single material system, marking a significant step forward in developing hardware-based neuromorphic computing.
